# Synthesis, structural characterization and antimycobacterial evaluation of several halogenated non-nitro benzothiazinones

**DOI:** 10.1007/s00044-021-02735-4

**Published:** 2021-06-10

**Authors:** Balungile Madikizela, Tamira Eckhardt, Richard Goddard, Adrian Richter, Anika Lins, Christoph Lehmann, Peter Imming, Rüdiger W. Seidel

**Affiliations:** 1 Institut für Pharmazie, Martin-Luther-Universität Halle-Wittenberg, Wolfgang-Langenbeck-Str. 4, Halle (Saale), 06120 Germany; 2 Phytomedicine Programme, Department of Paraclinical Sciences, University of Pretoria, Private Bag X04, Onderstepoort, 0110 South Africa; 3 Max-Planck-Institut für Kohlenforschung, Kaiser-Wilhelm-Platz 1, Mülheim an der Ruhr, 45470 Germany; 4 Department of Medicine and Department of Microbiology and Immunology, University of British Columbia, Vancouver, BC V6T 1Z3 Canada

**Keywords:** Benzothiazinones, Tuberculosis, Antimycobacterial evaluation, in vitro activity, Synthesis, Crystal structure

## Abstract

8-Nitro-1,3-benzothiazin-4-ones (BTZs), with BTZ043 and PBTZ169 as the most advanced compounds, represent a new class of potent antitubercular agents, which irreversibly inhibit decaprenylphosphoryl-β-d-ribose-2′-epimerase (DprE1), an enzyme crucial for cell wall synthesis in the pathogen *Mycobacterium tuberculosis*. Synthesis, structural characterization and in vitro testing against *Mycobacterium aurum* DSM 43999 and *M. tuberculosis* H_37_Rv of halogenated 2-(4-ethoxycarbonylpiperazin-1-yl)-1,3-benzothiazin-4-ones lacking a nitro group are reported. X-ray crystallography reveals that the structure of the BTZ scaffold can significantly deviate from planarity. In contrast to recent reports, the results of the present study indicate that further investigation of halogenated non-nitro BTZs for antitubercular activity is less than a promising approach.

## Introduction

Tuberculosis (TB) is an infectious disease, which is among the top ten causes of death worldwide. An estimated 1.2 million HIV-negative and 208,000 HIV-positive patients died of TB in 2019 [1]. TB is caused by *Mycobacterium tuberculosis* and typically manifests in the lungs (pulmonary TB) but can also affect other organs (extrapulmonary TB). Although about 85% of patients with TB can be cured with a drug regimen of 6 months [1], drug-resistant TB has become a threat to public health [2–4]. Moreover, disruptions of routine services for the management of TB due to lockdowns against SARS-CoV2 may cause a long-lasting increase in TB burdens in certain regions [5]. Novel anti-TB drugs are therefore needed to improve cure rates and to decrease the duration of TB treatments.

**Scheme 1:**
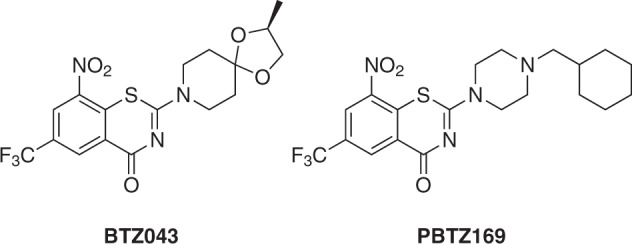
Chemical diagrams of BTZ043 and PBTZ169 (macozinone)

Both progression of late stage anti-TB drug candidates into the clinics and early drug discovery are vital to maintain a TB drug pipeline [6]. 1,3-Benzothiazin-4-ones (BTZs) are a promising class of new anti-TB drug candidates, which were first reported by Makarov et al. [7]. The as yet most advanced compounds BTZ043 and PBTZ169 (Scheme 1) exhibit nanomolar in vitro activity against *M. tuberculosis* and have reached clinical trials [6, 8]. It has been shown that 8-nitro-BTZs are suicide inhibitors of decaprenylphosphoryl-β-d-ribose 2′-epimerase (DprE1) [9], a mycobacterial enzyme that is crucial for cell wall synthesis and a known drug target [10, 11]. The drawbacks of nitro-aromatic anti-infective agents [12] such as BTZ043 and PBTZ169 are potential hepatotoxicity [13] and mutagenicity [14, 15]. Thus, efforts have been made to develop antitubercular BTZs in which the nitro group at C-8 of the BTZ scaffold has been replaced by e.g. a pyrrole [16], an azide [17] or a cyano group [18–20].

Recently, Nosova et al. published a series of fluorinated BTZs lacking a nitro group, for one of which, namely 5-fluoro-2-(4-ethoxycarbonylpiperazine-1-yl)-1,3-benzothiazin-4-one, a promising in vitro activity against *M. tuberculosis* H_37_Rv of 0.7 µg mL^−1^ (2 µM) was reported [21, 22]. Motivated by these findings, we synthesized and structurally characterized the aforementioned most active compound described by Nosova et al. and four related halogenated non-nitro BTZs and tested their in vitro activities against *Mycobacterium aurum* DSM 43999 and *M. tuberculosis* H_37_Rv. *M. aurum*, a fast-growing mycobacterial species with low pathogenicity [23], is considered a good model for *M. tuberculosis* [24, 25]. Herein, we discuss the molecular structures and results of antimycobacterial evaluation of five halogenated non-nitro BTZs.

## Results and discussion

### Synthesis

**Scheme 2:**
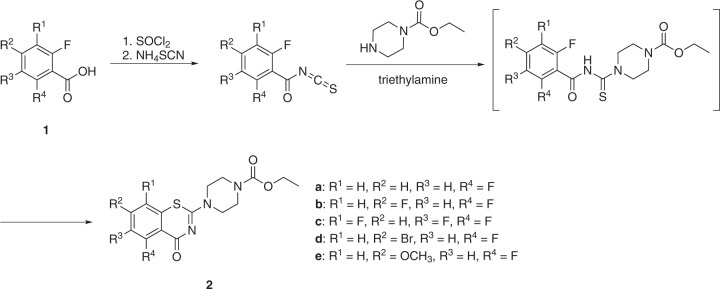
Synthesis of halogenated benzothiazinones **2a**–**e**

A variety of methods for the synthesis of BTZs have been described in the literature [26]. The halogenated non-nitro BTZs studied in this work were synthesized from *ortho*-fluorobenzoic acids (**1**) as starting materials (Scheme 2), in analogy to the previously reported synthesis of **2a** [21, 22]. The synthesis essentially followed Makarov’s original route to BTZs [27]. Treatment of **1** with thionyl chloride in toluene afforded the corresponding substituted benzoyl chlorides, which were reacted with ammonium thiocyanate in acetonitrile to yield the halogenated benzoyl isothiocyanates. Reaction with ethyl piperazine-1-carboxylate in acetonitrile gives the corresponding substituted thiourea intermediate products, which in general can be isolated in good yields [22]. In the work described here, the respective isothiocyanate and ethyl piperazine-1-carboxylate were treated in situ with triethylamine to obtain the halogenated BTZs **2** directly through an intramolecular heterocyclization reaction in a one-pot synthesis. Compounds **2a**-**e** were characterized by ^1^H and ^13^C NMR spectroscopy and high-resolution mass spectrometry (see Supplementary Material).

### Structural characterization

**Fig. 1:**
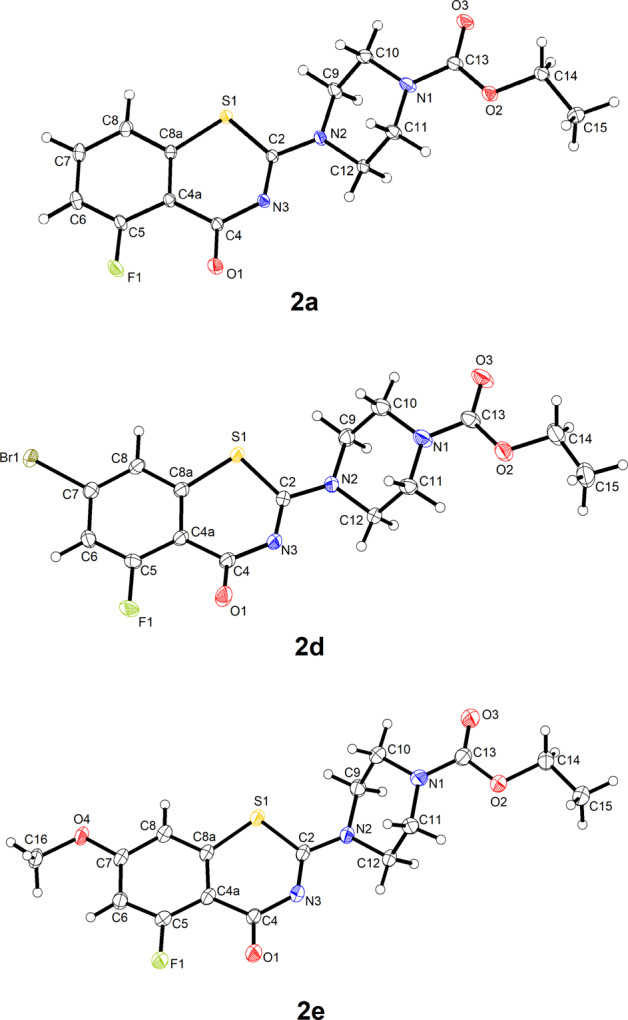
Molecular structures of **2a**, **2d** and **2e** in the crystal. Displacement ellipsoids are drawn at the 50% probability level. Hydrogen atoms are represented by small spheres of arbitrary radius. The solvate water molecule in **2a** · H_2_O is omitted for clarity

**Fig. 2:**
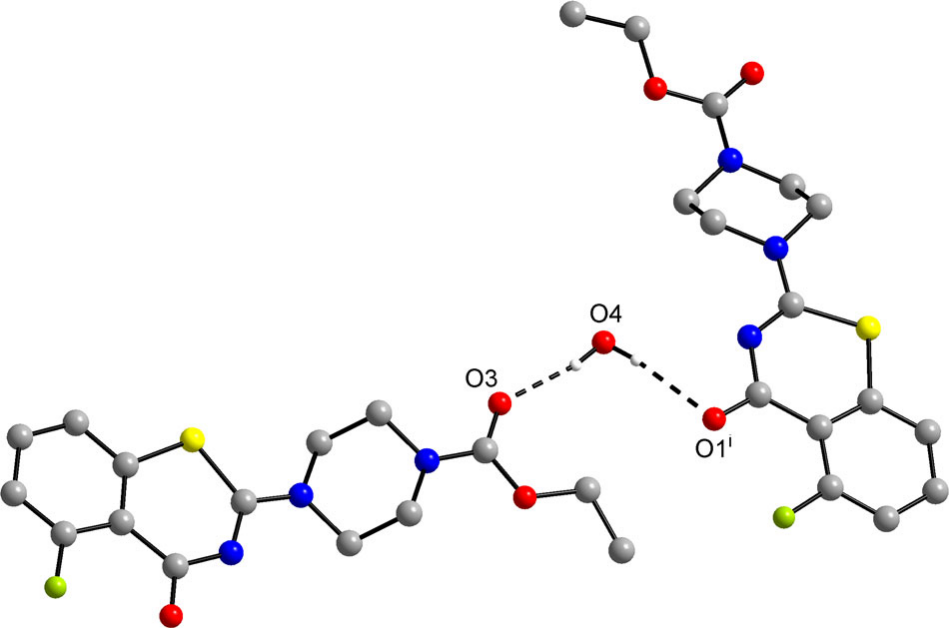
Hydrogen-bonded association between the solvate water molecule and two molecules of **2a** in the crystal structure of **2a** · H_2_O. Carbon-bound hydrogen atoms are omitted for clarity. *d*(O4···O3) = 2.8472(9) Å, <(O4−H4A···O3) = 169(1)°; *d*(O4···O1^i^) = 3.001(1) Å, <(O4−H4B···O1^i^) = 172(1)°. Symmetry code: (i) −x + 1, y + 1/2, −z + 3/2

The molecular structures of **2a**-﻿**e** were revealed by X-ray crystallography. **2a** · H_2_O, **2d** and **2e** crystallize with one BTZ molecule in the asymmetric unit (Z′ = 1). Figure 1 depicts the molecular structures. It should be noted that the space groups of these crystal structures are all centrosymmetric and thus the crystals contain the enantiomeric conformers of the molecular structures shown in Fig. 1. In **2a** · H_2_O and **2e**, the BTZ scaffold is virtually planar, whereas the 1,3-thiazin-4-one moiety deviates significantly from planarity in **2d**. O1 in **2d** is displaced from the mean plane through the condensed benzene ring by 0.515(3) Å. The piperazine six-membered ring adopts a low-energy chair conformation in the three structures. The structure at the piperazine nitrogen atoms N1 and N2 is virtually planar in each case, which can be ascribed to conjugation of the nitrogen lone pair with the carboxylate group and the BTZ π electron system, respectively. The carbamate group is virtually planar, as expected, and the ethyl group adopts an *anti* conformation about the O2−C14 bond. In **2e**, the methoxy group lies almost in the benzene ring plane, as expected [28], with a C6−C7−O4−C16 torsion angle of 2.1(3)°. The water molecule in **2a** · H_2_O acts as a hydrogen donor towards the carbonyl oxygen atom O3 of the carbamate group and O1 of the BTZ scaffold of an adjacent molecule (Fig. 2). Geometric parameters of the hydrogen bonds lie within the expected range for O−H···O=C hydrogen bonds [29]. Aside from the hydrogen-bonded water molecule in **2a** · H_2_O, the crystal structures appear to be governed by close packing. The packing index is 72.1% for **2a** · H_2_O, 70.5% for **2d** and 72.2% for **2e**, indicating a dense crystal packing [30]. Short interhalogen contacts are not encountered.

**Fig. 3:**
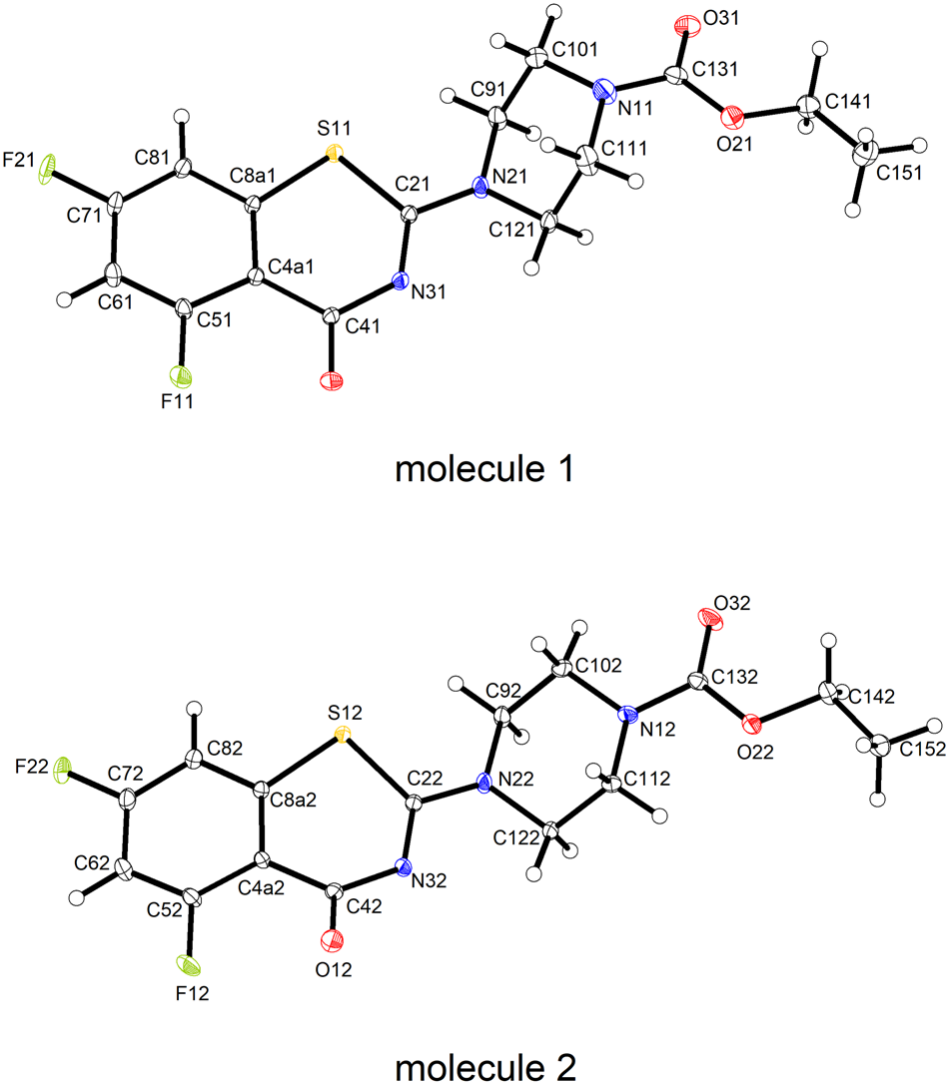
Displacement ellipsoid plots (50% probability level) of the two crystallographically unique molecules in **2b**. Hydrogen atoms are represented by small spheres of arbitrary radius

**Fig. 4:**
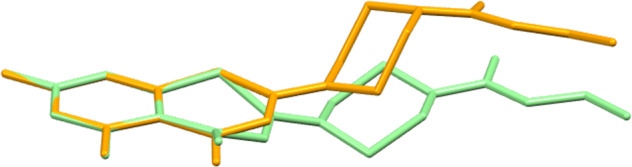
Structure overlay diagram of the benzene rings of the crystallographically unique molecules 1 (orange) and 2 (green) in **2b**. Hydrogen atoms are omitted for clarity

In contrast to **2a** · H_2_O, **2d** and **2e**, **2b** with fluorine substituents in the 5 and 7 positions of the BTZ scaffold crystallizes with two molecules in the asymmetric unit (Z′ = 2) [31]. Figure 3 depicts the two crystallographically unique molecules. Since the crystal structure is centrosymmetric, the enantiomeric conformers of both molecules are also present in the unit cell. In molecule 1, the BTZ scaffold is virtually planar as for **2a** · H_2_O and **2e**. In molecule 2, however, the 1,3-thiazin-4-one six-membered ring adopts a significant boat form with C22 and N32 located 0.46(2) and 0.41(2) Å, respectively, above the mean plane through the condensed benzene ring, and O12 lying 0.38(1) Å below this plane. A structure overlay diagram, shown in Fig. 4, illustrates the structural differences between the two crystallographically unique molecules. The ethyl piperazine-1-carboxylate moiety adopts the same conformation as in **2a** · H_2_O, **2d** and **2e**, as described above. In the crystal, the molecules are densely packed with a packing index of 72.3%.

**Fig. 5:**
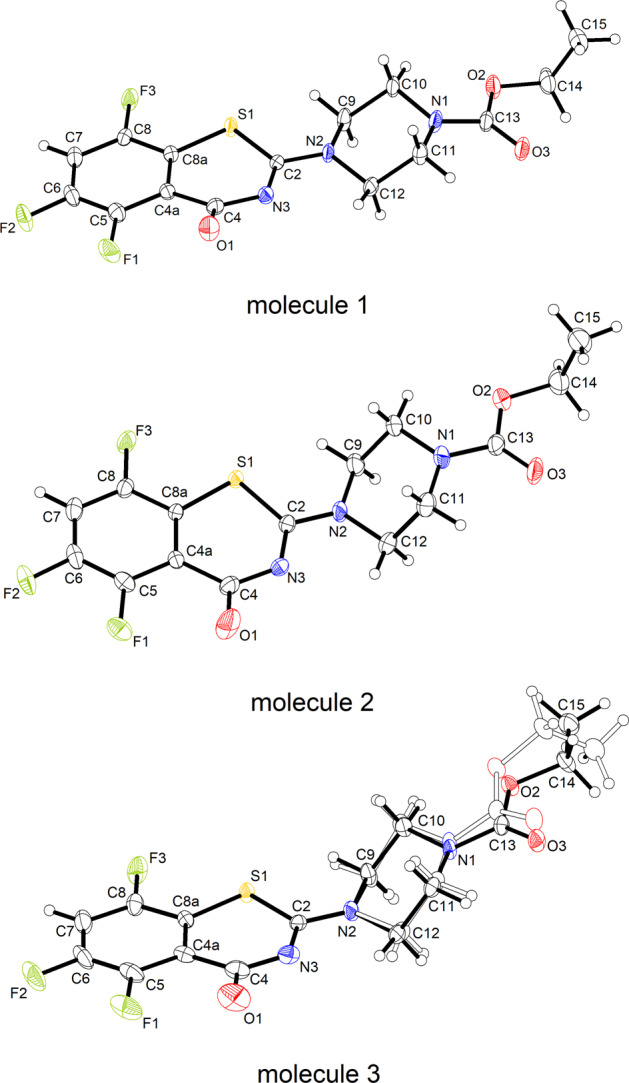
Displacement ellipsoid plots (50% probability level) of the three crystallographically unique molecules in **2c**. Hydrogen atoms are represented by small spheres of arbitrary radius. The minor disorder part (49 %) in molecule 3 is shown by empty ellipsoids

**Fig. 6:**
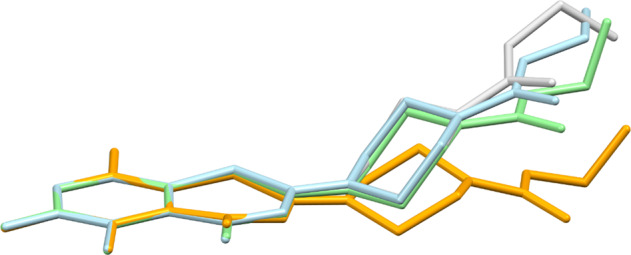
Structure overlay diagram of the benzene rings of the crystallographically unique molecules 1 (orange), 2 (green) and 3 (blue for the major and grey for the minor disorder component) in **2c**. Hydrogen atoms are omitted for clarity

Compound **2c** crystallizes with three molecules in the asymmetric unit (Z′ = 3) [31]. This crystal phase (space group *Cc*) was encountered at 100 K, 150 K and room temperature. Figure 5 depicts the structures of the three crystallographically unique molecules. With regard to the space group symmetry, the enantiomeric conformers are also present in the unit cell. Molecule 3 exhibits disorder of the ethyl piperazine-1-carboxylate moiety. In molecule 1, the benzothiazinone scaffold shows significant deviations from planarity with O1 displaced from the mean plane through the benzene ring by 0.488(6) Å. In molecules 2 and 3 the benzothiazinone scaffold is virtually planar. Figure 6 shows a structure overlay plot of the three unique molecules. As in **2a** · H_2_O, **2b**, **2d** and **2e**, the piperazine ring adopts a low-energy chair conformation in the three unique molecules in **2c**, but in contrast the ethyl group is tilted out of plane of the carbamate moiety [C−O−C−C torsion angles: −88.1(4), −90.5(5) and −85.3(8)°], with exception of the minor disorder component of molecule 3 where the C−O−C−C torsion angle is 173.0(7)°. Intermolecular short F···F contacts, with respect to the van der Waals radii [32], are observed between molecule 2 and molecule 3. It is worth noting that in the solid of **2c** there are short N−C−H···O=C intermolecular contacts (*D*···*A* distances < 3.3 Å) between the methylene groups of the piperazine moieties and carbonyl oxygen atoms of both the benzothiazone and the ester parts of all independent molecules, with the exception of the disordered ester in molecule, indicating that these contacts may be the result of an attractive N−C−H···O=C interaction.

Structural information on BTZs is still limited in the literature. Structures of the nitroso forms of BTZ043 (PDB code: 4F4Q) [33], related BTZs [34] and PBTZ169 (4NCR) [35] covalently bound to DprE1 can be found in the Protein Data Bank (PDB) [36]. In the crystal structure of the *M. tuberculosis* DprE1-PBTZ169 complex (4NCR; resolution: 1.9 Å), the covalently bound nitroso form of the piperazinyl-BTZ PBTZ169 (Scheme 1) exhibits a virtually planar BTZ scaffold, and the piperazine ring adopts chair conformation, similar to the structures of **2a**-**e**. A water molecule bridges the BTZ carbonyl oxygen atom and the backbone carbonyl oxygen atom of a leucine moiety via hydrogen bonding in 4NCR, which has some similarity to the hydrogen-bonding pattern in the crystal structure of **2a** · H_2_O (Fig. 2). In March 2021, only four small molecule structures of BTZs were available in the Cambridge Structural Database (CSD) [37], as revealed by a WebCSD search [38]. The crystal structure of PBTZ169 (CSD refcode: LOPXAS) was reported by Zhang and Aldrich [39] and that of its 8-CN analogue (WALHAW) very recently by Zhang et al. [18]. A virtually planar BTZ scaffold and a chair conformation of the piperazine ring are encountered in both structures. This is comparable with the conformations observed in **2a** · H_2_O, **2e** and molecule 1 in **2b**. To date, the crystal structures of two fluorinated non-nitro BTZs have been reported, namely 6,7,8-trifluoro-2-(thiomorpholin-4-yl)-1,3-benzothiazin-4-one (DOGXUV) [22] and 5-fluoro-2-(1-methyl-1H-pyrrol-2-yl)-1,3-benzothiazin-4-one (SOJGOQ) [40]. DOGXUV likewise exhibits a planar BTZ scaffold with the tethered thiomorpholin ring in a chair conformation. In SOJGOQ, the 1,3-thiazin-4-one moiety adopts a slight boat shape similar to **2d** and molecule 2 in **2b** but with the sulfur atom disordered over two positions. In contrast to **2a** · H_2_O, **2b**, **2d**, **2e** and SOJGOQ, intermolecular F···F contacts that are closer than the sum of the van der Waals radii [32] are found in DOGXUV, which may be classified as type I C−F···F−C interactions [41].

### Antimycobacterial evaluation

The antimycobacterial activity of halogenated non-nitro BTZs **2a-﻿e** was tested in vitro against *M. tuberculosis* H_37_Rv and *M. aurum* DSM 43999 (Table 1). BTZ043 was included as a reference compound, and the observed MIC_90_ value against *M. tuberculosis* H_37_Rv is consistent with the literature [7, 33]. Against *M. tuberculosis* H_37_Rv, **2d** inhibited growth at a concentration of 60 µM, whereas the other non-nitro BTZs studied did not show any antimycobacterial effect at the concentrations tested. In contrast to our results, Nosova et al. reported a MIC of 0.7 µg mL^−1^ (2 µM) for **2a** against *M. tuberculosis* H_37_Rv [21, 22]. We assume that major differences in the assay protocols used are responsible for this observation. Nosova et al. cultured *M. tuberculosis* H_37_Rv in Löwenstein-Jensen medium and evaluated the antimycobacterial effect of the test compounds by measuring the zone of growth retardation in test tubes, whereas our assay was carried out in 7H9 medium supplemented with 10% OADC and 0.05% polysorbate 80 using 96-well plates. For the assay system used in the present study, a *M. tuberculosis* H_37_Rv strain transformed with the pTEC27 plasmid [42, 43] for RFP expression was used and growth was analyzed after 7 days of incubation by fluorescence measurement [44, 45]. For MIC determination against *M. aurum* DSM 43999, a broth microdilution method with iodonitrotetrazolium chloride (INT) as indicator reagent was used. **2c**, **2d** and **2e** showed modest activity with MICs between 36 and 71 µM against *M. aurum* DSM 43999, whereas **2a** and **2b** do not exhibit activity against this mycobacterial strain.

**Table 1:** In vitro antimycobacterial activity of halogenated non-nitro BTZs 2a-e and the reference compound BTZ043

Compound	*M. tuberculosis* H_37_Rv MIC_90_/µM	*M. aurum* DSM 43999 MIC/µM
**2a**	>30	>100
**2b**	>100	>100
**2c**	>100	36
**2d**	60	48
**2e**	>100	71
BTZ043	0.003	0.008

In summary, antimycobacterial evaluation of **2a**-**e** did not reveal potent effects in vitro. Compared with 8-nitro-BTZs with nanomolar in vitro activity against *M. tuberculosis* [7, 46], the halogenated non-nitro congeners studied in the present work clearly display loss of activity. The presence of a nitro group at C-8 of the BTZ scaffold seems to be essential for the efficient, mechanism-based inhibition of DprE1 with high in vitro potency. Our findings thus are in line with previous studies that showed that 8-nitro BTZs bind covalently to DprE1 through linkage of their nitroso forms to the Cys387 residue in the FAD-binding domain [9, 33, 34, 47, 48]. It should be noted, however, that BTZs where the 8-nitro group was replaced by a pyrrole ring, and which are thought to act as non-covalent DprE1 inhibitors, showed similar in vitro activity but no efficacy in animal models [16].

## Conclusions

We synthesized and structurally characterized halogenated BTZs **2a-﻿e** lacking a nitro group at C-8 of the BTZ scaffold, and investigated their in vitro activities against *M. aurum* DSM 43999 and *M. tuberculosis* H_37_Rv. Structural characterization by X-ray crystallography revealed that the crystal packing can cause significant deviations from planarity in the BTZ moiety. The orientation of the ethyl piperazine-1-carboxylate moiety with respect to the BTZ plane gives rise to two enantiomeric conformers in the solid state in every case. The crystal structure of **2a** · H_2_O furthermore gives structural insight into hydrogen bonding acceptor properties of the BTZ molecule. In the absence of water of crystallization, the methylene groups of the piperazine ring appear to act as weak hydrogen bond donors to BTZ and carbamate carbonyl oxygen atoms. In vitro tests against *M. aurum* and *M. tuberculosis* did not reveal potent inhibitory effects, which lends support to the view that the 8-nitro group is essential for an efficient, mechanism-based inhibition of DprE1. In contrast to recent reports literature, our results thus indicate that further investigation of halogenated non-nitro BTZs for antitubercular activity is less than a promising approach.

## Experimental

### General

Starting materials were purchased and used as received. Solvents were distilled prior to use and stored over 4 Å molecular sieves. Column chromatography was carried out using Merck silica gel 60 (63–200 µm). Flash chromatography was performed on a puriFlash^®^ 430 instrument (Interchim, Montluçon, France). Prepacked columns with silica gel (30 μm) were used. The maximum compound load per column was 5% (m/m) of the silica gel quantity. The synthesis of BTZ043 is described elsewhere [27]. Melting points (uncorrected) were determined on a Boëtius hot-stage apparatus (VEB Kombinat, NAGEMA, Dresden, GDR). NMR spectra were recorded on an Agilent Technologies VNMRS 400 MHz and a Varian INOVA 500 NMR spectrometer. Chemical shifts are reported relative to the residual solvent signal (chloroform-*d*: δ_H_ = 7.26 ppm, δ_C_ = 77.16 ppm; methanol-*d*_4_: δ_H_ = 3.31 ppm, δ_C_ = 49.00 ppm; DMSO-*d*6 δ_H_ = 2.50 ppm, δ_C_ = 39.52 ppm). Abbreviations: s = singlet, bs = broad singlet, d = doublet, dd = doublet of doublets, m = multiplet. ESI high-resolution mass spectra (HRMS) were recorded on a Thermo Fisher Scientific LTQ Orbitrap XL mass spectrometer for **2b**, **2c** and **2e**, and on a Thermo Scientific Q Exactive^TM^ Plus Orbitrap mass spectrometer for **2a** and **2d**.

### Synthesis

#### General method for the preparation of the substituted benzoyl chloride precursors

The respective substituted benzoic acid was dissolved in toluene (ca. 20 mL per mmol) and two equivalents of thionyl chloride were added. After heating to reflux for 2 h, the solvent and excess thionyl chloride were removed in vacuum and the substituted benzoyl chloride thus obtained was used in the next synthetic step without purification.

#### 5-Fluoro-2-(4-ethoxycarbonylpiperazine-1-yl)-1,3-benzothiazin-4-one (**2a**)

The synthesis of **2a** can be found in the literature [22]. ^1^H NMR (400 MHz, DMSO-*d*_6_) δ 7.65 (td, *J* = 8.1, 5.0 Hz, 1H, aromatic CH), 7.46 (d, *J* = 8.0 Hz, 1H, aromatic CH), 7.29 (ddd, *J* = 11.2, 8.2, 0.9 Hz, 1H, aromatic CH), 4.08 (q, *J* = 7.1 Hz, 2H, ethyl CH_2_), 3.93–3.67 (m, 4H, piperazinyl CH_2_), 3.59–3.46 (m, 4H, piperazinyl CH_2_), 1.21 (t, *J* = 7.1 Hz, 3H, ethyl CH_3_). ^13^C NMR (126 MHz, DMSO-*d*_6_) δ 165.0 (d, *J*_C,F_ = 5.1 Hz), 161.8 (d, *J*_C,F_ = 262 Hz), 160.2, 154.5, 134.4, 133.6 (d, *J*_C,F_ = 10 Hz), 122.3 (d, *J*_C,F_ = 4 Hz), 116.3 (d, *J*_C,F_ = 23 Hz), 112.4 (d, *J*_C,F_ = 9 Hz), 61.0, 45.2, 42.6, 14.5 ppm. *m*/*z* [M + Na]^+^ calcd. for C_15_H_16_FN_3_NaO_3_S^+^ 360.07886, found 360.07878.

#### 5,7-Difluoro-2-(4-ethoxycarbonylpiperazine-1-yl)-1,3-benzothiazin-4-one (**2b**)

2,4,6-Trifluorobenzoyl chloride (5.14 mmol) was prepared by the general method and taken up in 2 mL of acetonitrile and a solution of ammonium thiocyanate (0.39 g, 5.14 mmol) in 15 mL of acetonitrile was added with stirring. The reaction mixture was stirred for 5 min at 40 °C and the ammonium chloride precipitate so formed was removed by filtration and ethyl piperazine-1-carboxylate (0.75 mL, 5.14 mmol) was added with stirring. After stirring for 3 h at room temperature, triethylamine (1.4 mL, 10.28 mmol) was added dropwise and the mixture was stirred overnight. Subsequently, the solvent was removed under reduced pressure and the crude product was purified by flash chromatography (ethyl acetate/heptane gradient). It was obtained as an off-white solid; mp 152–157 °C; yield: 0.56 mg (1.58 mmol, 31 %). ^1^H NMR (400 MHz, MeOH-*d*_4_) δ 7.28 (ddd, *J* = 8.3, 2.5 Hz, 1.5 Hz, 1H, aromatic CH), 7.13 (ddd, *J* = 11.3, 9.0, 2.5 Hz, 1H, aromatic CH), 4.17 (q, *J* = 7.1 Hz, 2H, ethyl CH_2_), 4.06–3.74 (m, 4H, piperazinyl CH_2_), 3.71–3.56 (m, 4H, piperazinyl CH_2_), 1.28 (t, *J* = 7.1 Hz, 3H, ethyl CH_3_) ppm. ^13^C NMR (101 MHz, MeOH-*d*_4_) δ 168.3 (d, *J*_C,F_ = 5 Hz), 167.0–166.6 (m), 164.1 (dd, *J*_C,F_ = 23, 13 Hz), 162.9, 157.0, 139.0 (dd, *J*_C,F_ = 12 Hz, 2 Hz), 110.2 (dd, *J*_C,F_ = 26 Hz), 110.2–110.1 (m), 106.4 (dd, *J*_C,F_ = 27, 26 Hz), 63.1, 46.9, 44.0, 14.9 ppm. HRMS(ESI^+^): *m*/*z* [M + Na]^+^ calcd. for C_15_H_15_F_2_N_3_NaO_3_S^+^ 378.0695, found 378.0692.

#### 5,6,8-Trifluoro-2-(4-ethoxycarbonylpiperazine-1-yl)-1,3-benzothiazin-4-one (**2c**)

Compound **2c** was synthesized analogously to **2b** from 2,3,5,6-tetrafluorobenzoyl chloride (4.71 mmol). In addition to the target compound **2c**, the thiourea intermediate product (see Scheme 2) was isolated by flash chromatography. It was taken up with 5 mL of dimethylformamide and treated with 2.3 mL of triethylamine. After stirring overnight and heating to 70 °C for 2 h, additional **2c** was isolated after removal of the solvent and recrystallization from isopropanol. It was obtained as an off-white solid; mp 192–197 °C; yield: 0.62 g (1.66 mmol, 35 %). ^1^H NMR (500 MHz, chloroform-*d*) δ 7.22 (td, *J* = 9.1, 5.9 Hz, 1H, aromatic CH), 4.18 (q, *J* = 7.1 Hz, 2H, ethyl CH_2_), 3.89 (bs, 4H, piperazinyl CH_2_), 3.64–3.52 (m, 4H, piperazinyl CH_2_), 1.28 (t, *J* = 7.1 Hz, 3H, ethyl CH_3_). ^13^C NMR (126 MHz, chloroform-*d*) δ 165.1, 160.1, 155.3, 152.0 (ddd, *J*_C,F_ = 246, 10, 5 Hz), 149.7 (ddd, *J*_C,F_ = 254, 15, 12 Hz), 148.1 (ddd, *J*_C,F_ = 265, 14, 4 Hz), 116.8 (dd, *J*_C,F_ = 18, 4 Hz), 114.6 (dd, *J*_C,F_ = 7, 2 Hz), 108.6 (dd, *J*_C,F_ = 25, 23 Hz), 62.2, 46.1, 43.2, 14.7. HRMS(ESI^+^): *m*/*z* [M + H]^+^ calcd. for C_15_H_15_F_3_N_3_O_3_S^+^ 374.0781, found 374.0780, [M + Na]^+^ calcd. for C_15_H_14_F_3_O_3_N_3_NaS^+^ 396.0601, found 396.0603.

#### 7-Bromo-5-fluoro-2-(4-ethoxycarbonylpiperazine-1-yl)-1,3-benzothiazin-4-one (**2d**)

Compound **2d** was synthesized analogously to **2b** from 4-bromo-2,6-difluorobenzoyl chloride but heated to reflux overnight. It was obtained as an off-white solid; mp 225–227°; yield: 0.51 g (1.23 mmol, 29 %). ^1^H NMR (400 MHz, chloroform-*d*) δ 7.34–7.27 (m, 2H, aromatic CH), 4.18 (q, *J* = 7.1 Hz, 2H, ethyl CH_2_), 4.00–3.73 (m, 4H, piperazinyl CH_2_), 3.66–3.54 (m, 4H, piperazinyl CH_2_), 1.28 (t, *J* = 7.1 Hz, 3H, ethyl CH_3_). ^13^C NMR (126 MHz, chloroform-*d*) δ 165.8 (d, *J*_C,F_ = 5 Hz), 162.4 (d, *J*_C,F_ = 270.4 Hz), 160.0, 155.1, 136.1, 126.1 (d, *J*_C,F_ = 11 Hz), 124.1 (d, *J*_C,F_ = 5 Hz), 120.1 (d, *J*_C,F_ = 26 Hz), 62.0, 45.8, 43.1, 14.6. HRMS(ESI^+^): *m*/*z* [M + Na]^+^ calcd. for C_15_H_15_BrFN_3_NaO_3_S^+^: 437.98939, found 437.98965.

#### 5-Fluoro-7-methoxy-2-(4-ethoxycarbonylpiperazine-1-yl)-1,3-benzothiazin-4-one (**2e**)

Compound **2e** was synthesized analogously to **2b** from 2,6-difluoro-4-methoxybenzoyl chloride (5.32 mmol) but heated to reflux overnight. It was obtained as a pale brown solid; mp 218–221 °C; yield: 0.32 g (0.87 mmol, 16 %). ^1^H NMR (400 MHz, chloroform-*d*) δ 6.67 (dd, *J* = 12.2, 2.5 Hz, 1H, aromatic CH), 6.63 (dd, *J* = 2.5, 1.1 Hz, 1H, aromatic CH), 4.18 (q, *J* = 7.1 Hz, 2H, ethyl CH_2_), 4.03–3.69 (m, 7H, piperazinyl CH_2_ and methoxy CH_3_), 3.64–3.55 (m, 4H, piperazinyl CH_2_), 1.29 (t, *J* = 7.1 Hz, 3H, ethyl CH_3_). ^13^C NMR (101 MHz, chloroform-*d*) δ 165.7 (d, *J*_C,F_ = 5 Hz), 163.5 (d, *J*_C,F_ = 266 Hz), 161.7 (d, *J*_C,F_ = 13 Hz), 159.5, 154.4, 135.1 (d, *J*_C,F_ = 3 Hz), 105.5 (d, *J*_C,F_ = 4 Hz), 105.3 (d, *J*_C,F_ = 10 Hz), 102.5 (d, *J*_C,F_ = 27 Hz), 61.1, 55.1, 44.8, 42.3, 13.8. HRMS(ESI^+^): *m*/*z* [M + H] calcd. for C_16_H_19_FN_3_O_4_S^+^ 368.1075, found 368.1079, [M + Na]^+^ calcd. for C_16_H_18_FN_3_NaO_4_S^+^ 390.0895, found 390.0893.

### X-ray crystallography

Crystals suitable for single-crystal X-ray diffraction were grown from dimethylformamide for **2a** · H_2_O, toluene for **2b**, acetone for **2c** and methanol for **2d** and **2e**. Diffraction data were measured at the P11 beamline at PETRA III at DESY [49, 50] with a Pilatus 6 M detector [51] for **2a** · H_2_O, **2b** and **2e**, on a Bruker AXS Kappa Mach3 APEXII diffractometer equipped with Incoatec microfocus source for **2c**, and on a Bruker AXS Kappa Mach3 APEXII diffractometer equipped with a FR591 Cu rotating anode X-ray source for **2d**. The data were processed with XDS [52] for **2a** · H_2_O**, 2b** and **2e** and SAINT [53] for **2c** and **2d**. For **2c** and **2d**, an absorption correction by face-indexed Gaussian integration was applied with SADABS [54]. The crystal structures were solved with SHELXT [55] and refined with SHELXL-2018/3 [56]. **2a** · H_2_O, **2b** and **2d** were refined using aspherical scattering factors [57], except for the water oxygen atom O4 in **2a** · H_2_O and Br1 and C7 in **2d**. Disorder of an ethyl piperazine-1-carboxylate moiety in **2c** was described by a split atom model using EADP constraints for respective atoms in the parts. Refinement of the ratio of occupancies by means of a free variable yielded 0.507(3):0.493(3). A Flack *x* parameter of 0.016(18) for **2c** was determined using 6035 quotients [(I+) − (I−)]/[(I+) + (I−)] (Parsons’ method) [58]. The crystal of **2c** studied was partially non-merohedrally twinned [−1 0 0 0 −1 0 0.75 0 1] with a refined minor component of 0.0279(6). In **2a** · H_2_O and **2b**, hydrogen atom positions and *U*_iso_(H) values were refined freely. In **2d**, hydrogen atom positions were refined and *U*_iso_(H) values were set 1.2 *U*_eq_(C) (1.5 for methyl groups). In **2c** and **2e**, hydrogen atoms were placed in geometrically calculated positions and refined using an appropriate riding model and *U*_iso_(H) = 1.2 *U*_eq_(C) (1.5 for methyl groups). Torsion angles of methyl groups in **2c** and **2e** were initially determined *via* circular Fourier syntheses and subsequently refined while maintaining tetrahedral angles at the carbon atom. Structure pictures were drawn with Diamond [59] and Mercury [60].

#### Crystal data for **2a** · H_2_O

C_15_H_18_FN_3_O_4_S, *M*_r_ = 355.38, *T* = 100(2) K, *λ* = 0.6199 Å, monoclinic, space group *P*2_1_/*c*, *a* = 10.659(2), *b* = 21.099(4), *c* = 7.4526(15) Å, *β* = 107.77(3)°, *V* = 1596.2(6) Å^3^, *Z* = 4, *ρ*_calc_ = 1.479 mg m^−3^, *μ* = 0.167 mm^−1^, *F*(000) = 744, crystal size 0.136 × 0.035 × 0.013 mm, *θ* range 1.68–27.00°, reflections collected/unique 33425/5016 (*R*_int_ = 0.0293), 292 parameters, *S* = 1.071, *R*1 [*I* > 2*σ*(*I*)] = 0.0249, *wR*2 = 0.0704, *Δρ*_max_/*Δρ*_min_ = 0.29/−0.34 eÅ^–3^.

#### Crystal data for **2b**

C_15_H_15_F_2_N_3_O_3_S, *M*_r_ = 355.36, *T* = 100(2) K, *λ* = 0.6199 Å, triclinic, space group *P*-1, *a* = 10.305(2), *b* = 12.616(3), *c* = 13.567(3) Å, *α* = 112.19(3), *β* = 102.29(3), *γ* = 100.93(3)°, *V* = 1523.1(6) Å^3^, *Z* = 4, *ρ*_calc_ = 1.550 mg m^−3^, *μ* = 0.178 mm^−1^, *F*(000) = 736, crystal size 0.087 × 0.052 × 0.043 mm, *θ* range 1.49–27.00°, reflections collected/unique 65202/10012 (*R*_int_ = 0.0349), 556 parameters, *S* = 1.034, *R*1 [*I* > 2*σ*(*I*)] = 0.0248, *wR*2 = 0.0682, *Δρ*_max_/*Δρ*_min_ = 0.40/−0.36 eÅ^–3^.

#### Crystal data for **2c**

C_15_H_14_F_3_N_3_O_3_S, *M*_r_ = 373.35, *T* = 150(2) K, *λ* = 0.71073 Å, monoclinic, space group *Cc*, *a* = 8.0599(3), *b* = 27.0465(11), *c* = 21.2617(8) Å, *β* = 98.159(2)°, *V* = 4588.0(3) Å^3^, *Z* = 12, *ρ*_calc_ = 1.622 mg m^−3^, *μ* = 0.268 mm^−1^, *F*(000) = 2304, crystal size 0.094 × 0.035 × 0.024 mm, *θ* range 0.97–33.20°, reflections collected/unique 85259/17444 (*R*_int_ = 0.0369), 712 parameters, 2 restraints, *S* = 1.052, *R*1 [*I* > 2*σ*(*I*)] = 0.0484, *wR*2 = 0.1319, *Δρ*_max_/*Δρ*_min_ = 0.78/−0.47 eÅ^–3^.

#### Crystal data for **2d**

C_15_H_15_BrFN_3_O_3_S, *M*_r_ = 416.27, *T* = 100(2) K *λ* = 1.54178 Å, monoclinic, space group *P*2_1_/*c*, *a* = 8.3506(2), *b* = 22.7598(5), *c* = 9.2142(2) Å, *β* = 110.481(1)°, *V* = 1640.53(6) Å^3^, *Z* = 4, *ρ*_calc_ = 1.685 mg m^−3^, *μ* = 4.890 mm^−1^, *F*(000) = 840, crystal size 0.150 × 0.133 × 0.051 mm, *θ* range = 5.48–72.14°, reflections collected/unique = 25434/2970 (*R*_int_ = 0.0406), 265 parameters, *S* = 1.084, *R*1 [*I* > 2*σ*(*I*)] = 0.0219, *wR*2 = 0.0527, *Δρ*_max_/*Δρ*_min_ = 0.42/−0.50 eÅ^–3^.

#### Crystal data for **2e**

C_16_H_18_FN_3_O_4_S, *M*_r_ = 367.39, *T* = 100(2) K, *λ* = 0.6199 Å, monoclinic, space group *P*2_1_/*c*, *a* = 10.525(2), *b* = 21.450(4), *c* = 7.6170(15) Å, *β* = 109.53(3)°, *V* = 1620.7(6) Å^3^, *Z* = 4, *ρ*_calc_ = 1.506 mg m^−3^, *μ* = 0.166 mm^−1^, *F*(000) = 768, crystal size 0.084 × 0.011 × 0.005 mm, *θ* range 1.66–26.94°, reflections collected/unique 32985/5208 (*R*_int_ = 0.1033), 228 parameters, *S* = 1.029, *R*1 [*I* > 2*σ*(*I*)] = 0.0649, *wR*2 = 0.1878, *Δρ*_max_/*Δρ*_min_ = 0.87/−0.38 eÅ^–3^.

### Microbiological assays

The mycobacterial strains were routinely grown in 7H9 broth (Difco Middlebrook) supplemented with 10% (v/v) OADC (5 % bovine albumin fraction, 2 % dextrose, 0.004 % catalase, 0.05 % oleic acid and 0.8 % sodium chloride solution) and 0.05% (v/v) polysorbate 80 at 37 °C in standing cultures. Hygromycin B was added to the medium at a final concentration of 50 µg mL^−1^ for *M. tuberculosis* H_37_Rv pTEC27.

For the antimycobacterial activity assay against *M. tuberculosis* H_37_Rv (harbouring RFP expressing pTEC27 plasmid; the plasmid confers resistance to hygromycin), MIC_**90**_ values were determined by the broth microdilution method using flat-bottom 96-well Corning Costar plates. The highest concentration tested for each compound was 200 µg mL^−1^. Each well with the test compound and 7H9 medium supplemented with 10% OADC, 0.05% polysorbate 80 and hygromycin (50 µg mL^−1^) was then diluted twofold in a ten-point serial dilution. The concentration of the inoculum of 5 × 10^5^ cells mL^−1^ (OD_600_, 0.1 = 0.33 × 10^8^ CFU mL^−1^) was prepared from a starting inoculum that was diluted from a preculture at the mid-log phase (OD_600_, 0.3 to 0.7). In each plate a negative control (1 % DMSO) and a positive control (100 µg mL^−1^ gentamicin) were included. Subsequently, 100 µL of the bacterial inoculum were added to each well to give a final volume of 200 μL. The plates were then sealed with parafilm, placed in a container with moist tissue and incubated for six days at 37 °C. After incubation the fluorescence intensity of each well was measured with a BioTek Synergy H4 plate reader (λ_ex_ = 530 nm, λ_em_ = 590 nm) and the MIC_90_ value was calculated as the concentration of the compound that caused more than 90% growth reduction. The determination was done in duplicate. The percentage of growth inhibition was calculated using the equation:

$${\mathrm{\% }}\,{\mathrm{growth}}\,{\mathrm{inhibition}} = - 100\,\% \times \frac{{{\mathrm{OD}}_{590}\left( {{\mathrm{sample}}} \right) - {\mathrm{OD}}_{590}\left( {{\mathrm{DMSO}}} \right)}}{{{\mathrm{OD}}_{590}\left( {{\mathrm{DMSO}}} \right) - {\mathrm{OD}}_{590}\left( {{\mathrm{gentamicin}}} \right)}}$$

Controls were used to monitor the assay quality through determination of the *Z*′ score. The *Z*′ factor was calculated as follows:

$$Z^\prime = 1 - \frac{{3\left( {{\mathrm{SD}}_{{\mathrm{gentamicin}}} + {\mathrm{SD}}_{{\mathrm{DMSO}}}} \right)}}{{M_{{\mathrm{gentamicin}}} - M_{{\mathrm{DMSO}}}}}$$

(SD = standard deviation, M = mean)

Against *M. aurum* DSM 43999, MIC values were determined using the broth microdilution method in 96-well flat-bottom tissue culture plates (Sarstedt, 83.3924.500), following a slightly modified literature procedure [61]. The test samples were dissolved in 10% DMSO at a concentration of 1 mg mL^−1^. In all the wells of a 96-well microtiter plate, 100 μL of OADC supplemented Middlebrook broth were added followed by addition of 100 μL of each sample in the first three wells. Broth and sample were mixed with the pipette and serially diluted down the wells. The highest concentration of the compounds tested was 250 μg mL^−1^.

The positive controls used in this study were ciprofloxacin, isoniazid, rifampicin and triclosan at a concentration of 5 mg mL^−1^. Broth only, 10% DMSO and *M. aurum* DSM 43999 only wells were used as the negative controls. *M. aurum* DSM 43999 suspension (100 μL; adjusted to match McFarland standard 0.5 [62]) was added to all the wells, except the broth only wells and incubated for 72 h at 37 °C. INT from Sigma-Aldrich, Germany (40 μL, 0.2 mg mL^−1^) was added to one column with the *M. aurum* DSM 43999 control and incubated for 30 min to one hour. When there was a colour change from clear to pink, the INT was added to all the wells and incubated for 30 min to 1 h. The changing of the clear INT solution to a pink colour means that there is a bacterial growth in the well. Thus, the MIC is defined as the lowest concentration of sample where the colour of the INT remains still clear.

## Supplementary information

Supplementary Information

## Data Availability

NMR and MS spectra are depicted in the Supplementary Material. CCDC 2074617-2074621 contain the supplementary crystallographic data for this paper. These data can be obtained free of charge from the Cambridge Crystallographic Data Centre via www.ccdc.cam.ac.uk/structures.
