# Supplementary material for: Synthesis, structural characterization and antimycobacterial evaluation of several halogenated non-nitro benzothiazinones
Source: Med Chem Res. 2021 Jun 10;30(8):1523–33. doi: 10.1007/s00044-021-02735-4 (PMC8192043; doi:10.1007/s00044-021-02735-4)
Supplement: Supplementary file 1 — Supplementary Information [file 44_2021_2735_MOESM1_ESM.pdf]

Supporting Information for

Synthesis, structural characterization and antimycobacterial evaluation of several halogenated non-nitro benzothiazinones

Balungile Madikizela, Tamira Eckhardt, Richard Goddard, Adrian Richter, Anika Lins, Christoph Lehmann, Peter Imming, Rüdiger W. Seidel

Content

- **Figure S1** ^{1}H NMR spectrum of **2a** in DMSO-*d*6.
- **Figure S2** ^{13}C{^{1}H} NMR spectrum of **2a** in DMSO-*d*6.
- **Figure S3** HRMS(ESI^{+}) analysis of **2a** in methanol.
- **Figure S4** ^{1}H NMR spectrum of **2b** in MeOH-*d*4.
- **Figure S5** ^{13}C{^{1}H} NMR spectrum of **2b** in MeOH-*d*4.
- **Figure S6** HRMS(ESI^{+}) spectrum of **2b** in methanol.
- **Figure S7** ^{1}H NMR spectrum of **2c** in chloroform-*d*.
- **Figure S8** ^{13}C{^{1}H} NMR spectrum of **2c** in chloroform-*d*.
- **Figure S9** HRMS(ESI^{+}) spectrum of **2c** in methanol.
- **Figure S10** ^{1}H NMR spectrum of **2d** in chloroform-*d*.
- **Figure S11** ^{13}C{^{1}H} NMR spectrum of **2d** in chloroform-*d*.
- **Figure S12** HRMS(ESI^{+}) analysis of **2d** in methanol.
- **Figure S13** ^{1}H NMR spectrum of **2e** in chloroform-*d*.
- **Figure S14** ^{13}C{^{1}H} NMR spectrum of **2e** in chloroform-*d*.
- **Figure S15** HRMS(ESI^{+}) spectrum of **2e** in methanol.

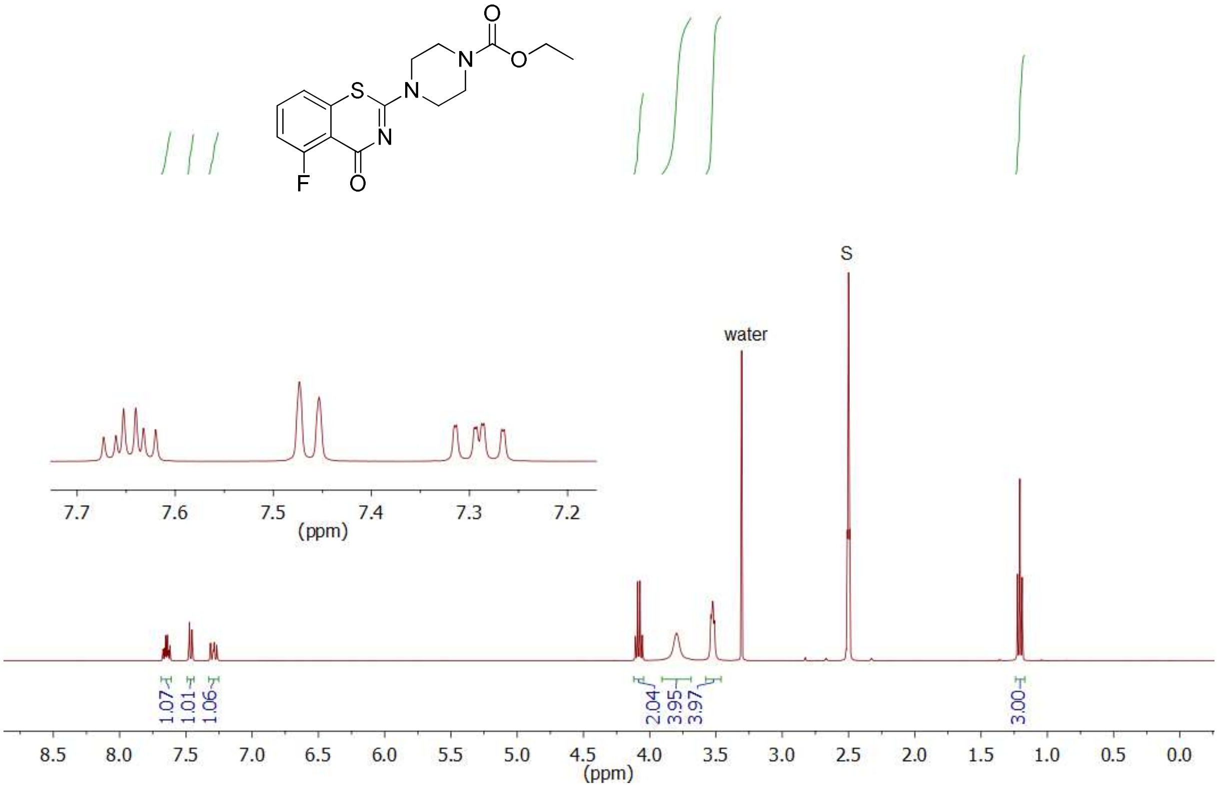

Figure S1 ^{1}H NMR spectrum of **2a** in DMSO-*d6*. S denotes the solvent peak.

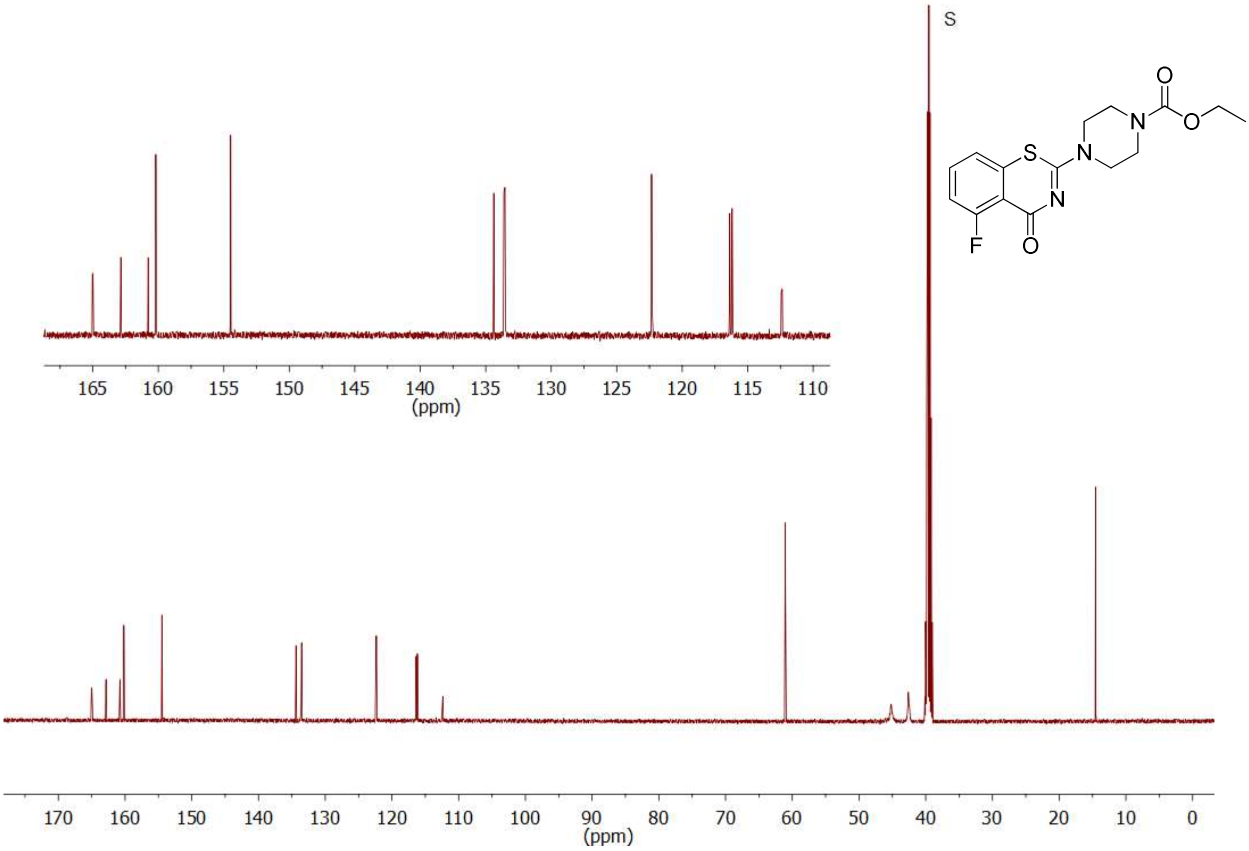

Figure S2 $^{13}C\{^{1}H\}$ NMR spectrum of **2a** in DMSO-*d6*. S denotes the solvent peak.

electrospray-ionization (Sol.: CH3OH) pos. ions

molecular weight 337 et. al. possible

characteristical ions
338 = [337 + H]+
360 = [337 + Na]+
675 = [2*337 + H]+
697 = [2*337 + Na]+

characteristical ions (doubly charged)
357 = [2*337 + Ca]2+
525 = [3*337 + Ca]2+

additional characteristical ions
363

additional characteristical ions (doubly charged)
234, 261

10.02.2021
File: 151617a-00.RAW

Analyse: GOD-GB-119-01
LMN: Goddard,Richard

Ionisierung: ESIpos
Lösungsmittel:CH3OH
Spektrometer: Exactive

Auswerter: Kampen (2242)

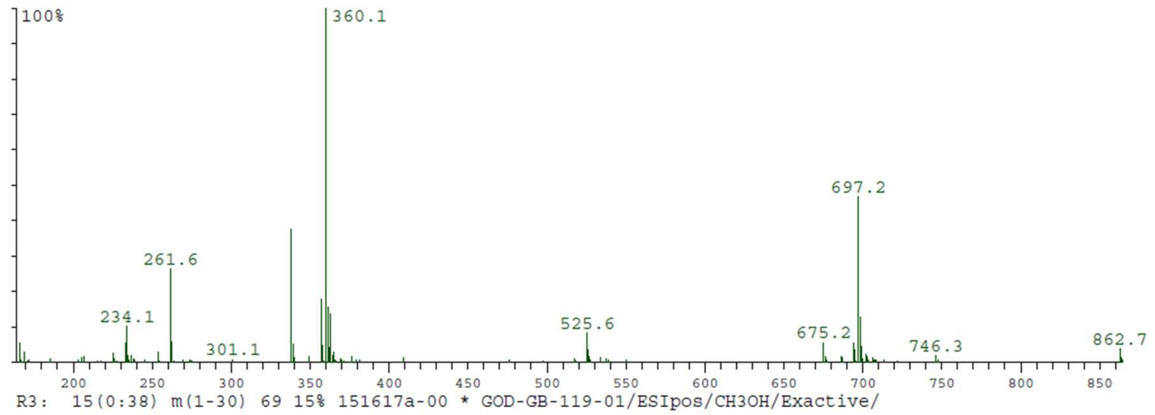

Mass to be matched (m/z): 360.078780 Charge: 1

Mass Tolerance: ±0.002500
Restriction of atom numbers:

C	H	F	N	O	S	Na
1-100	1-100	max 1	1-3	max 10	max 1	1-1

Number of calculated Formulas: 6

Formula	Diff.(ppm)	theor. m/z
C15 H16 F1 N3 O3 S1 Na1	0.23	360.078861
C26 H11 N1 Na1	-1.15	360.078368
C23 H12 F1 N1 O1 Na1	2.03	360.079511
C18 H15 N3 O2 S1 Na1	-2.95	360.077718
C11 H17 N2 O10 Na1	-3.44	360.077541
C14 H15 N3 O7 Na1	4.00	360.080220

Datum 10.02.2021
Analyse: 151617b-00

Sigel: GOD-GB-119-01
LMN: Goddard,Richard

Messung: HRMS
Methode: ESIpos
Lösungsmittel: CH3OH
Spektrometer: Exactive

Auswerter: Kampen (2242)

Suggestion:
C15H16F1N3O3S1 MW 337

characteristical ion
360 = [337 + Na]+

Figure S3 HRMS(ESI^{+}) analysis of **2a** in methanol.

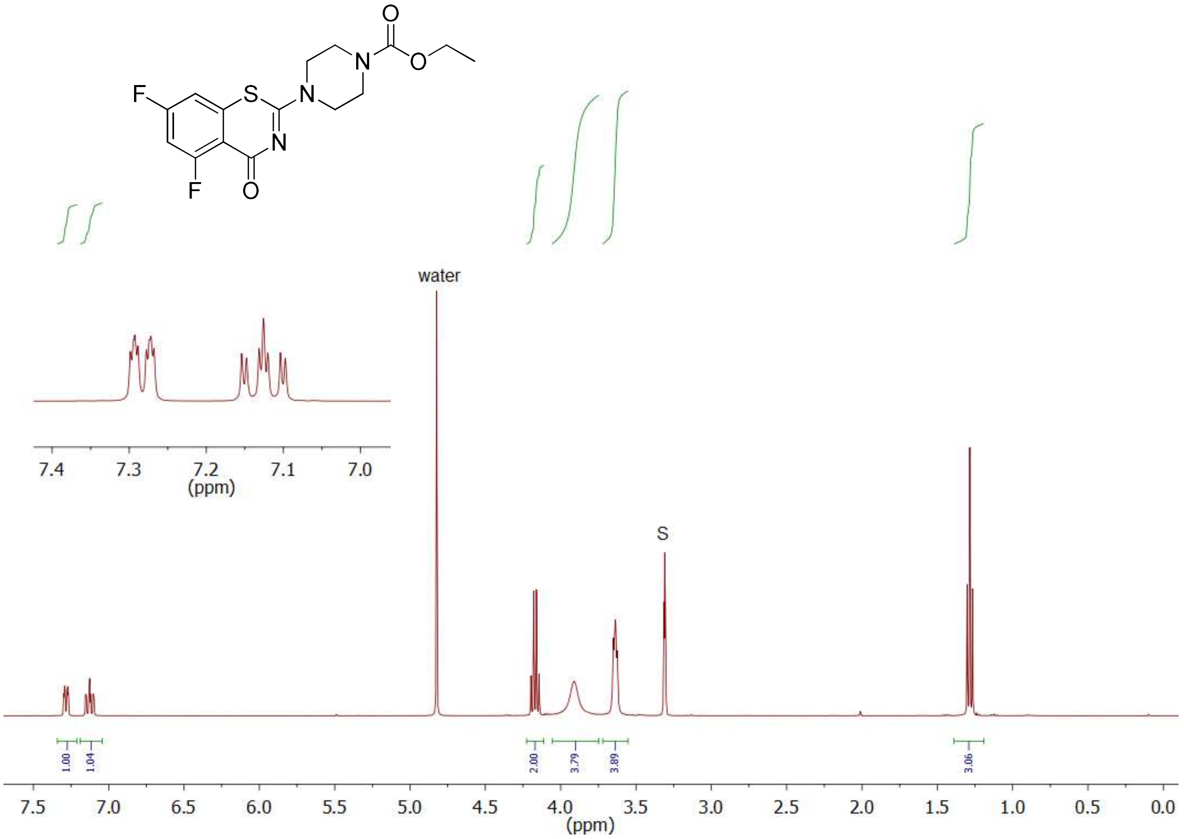

Figure S4 ^{1}H NMR spectrum of **2b** in MeOH-*d*4. S denotes the solvent peak.

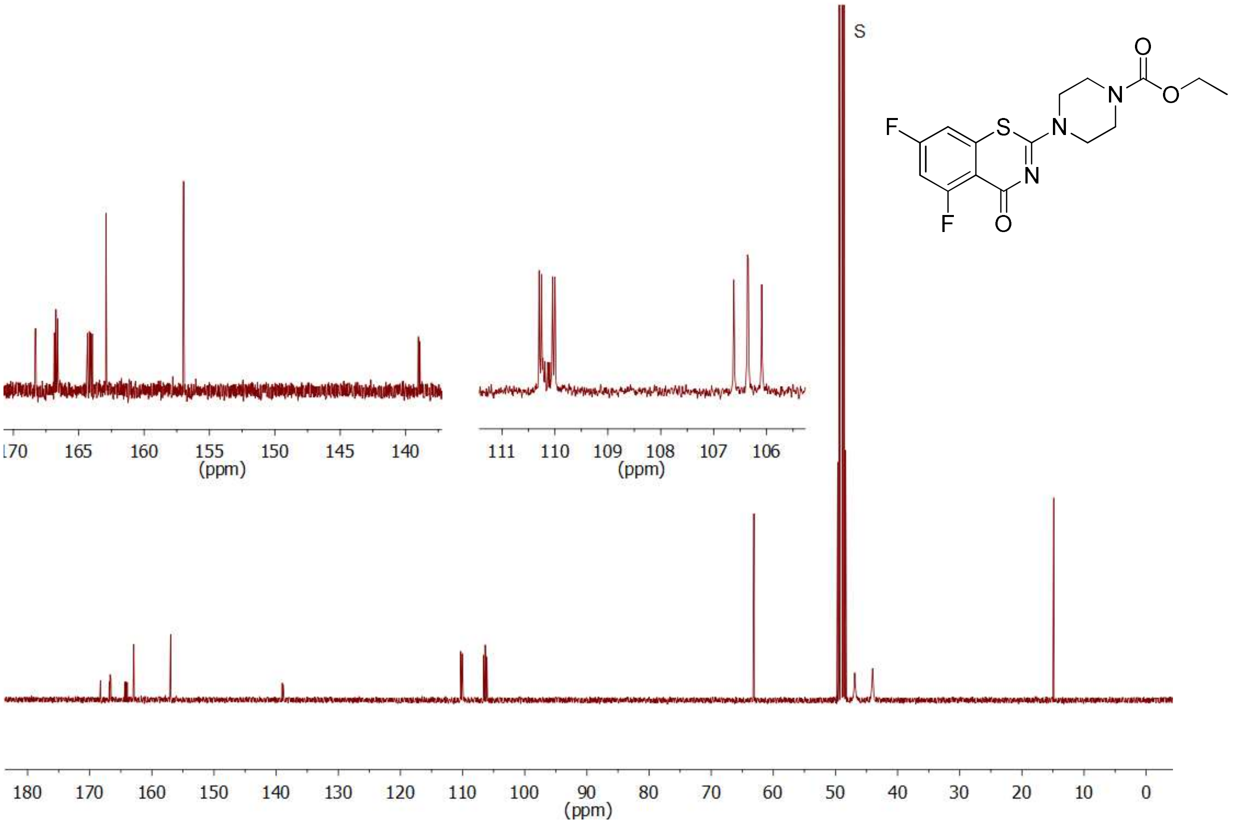

Figure S5 ^{13}C{^{1}H} NMR spectrum of **2b** in MeOH-*d*4. S denotes the solvent peak.

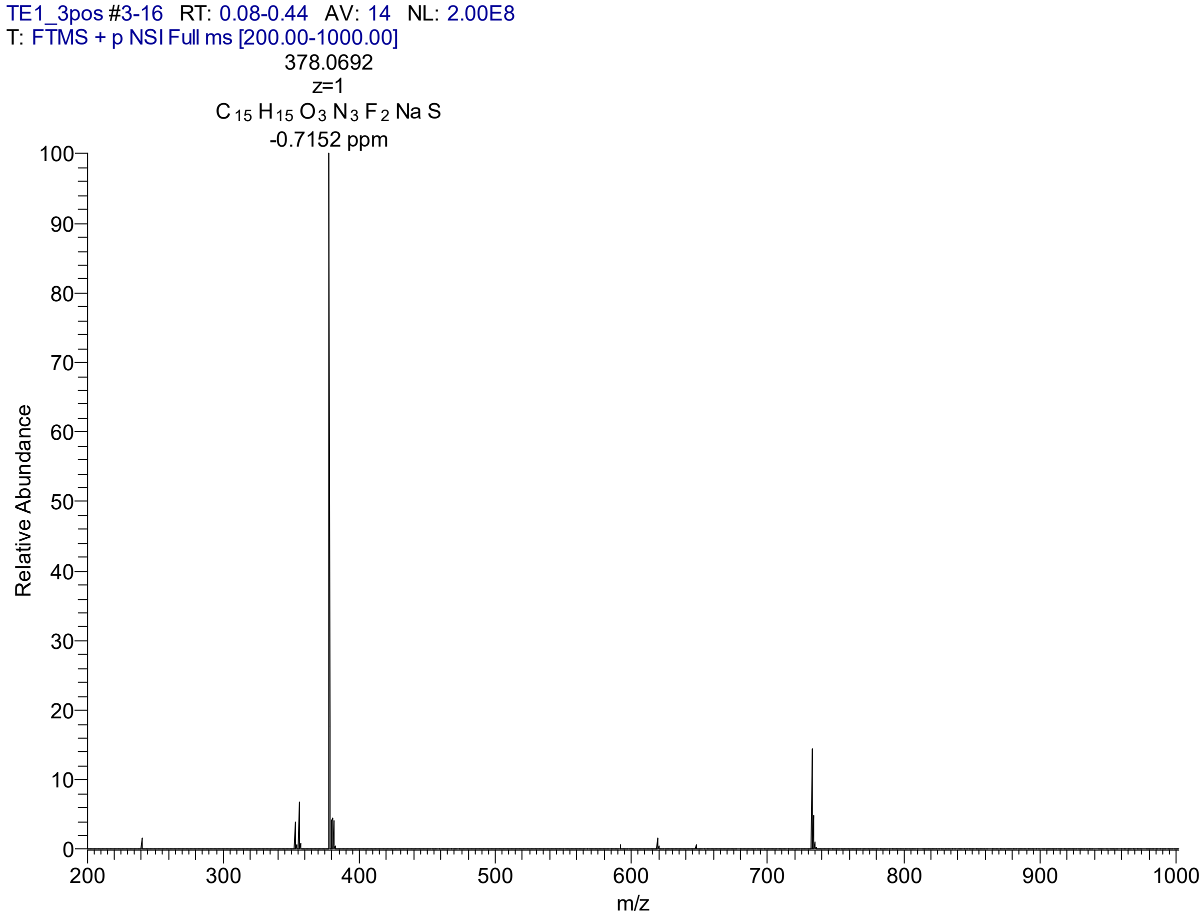

Figure S6 HRMS(ESI$^+$) spectrum of **2b** in methanol.

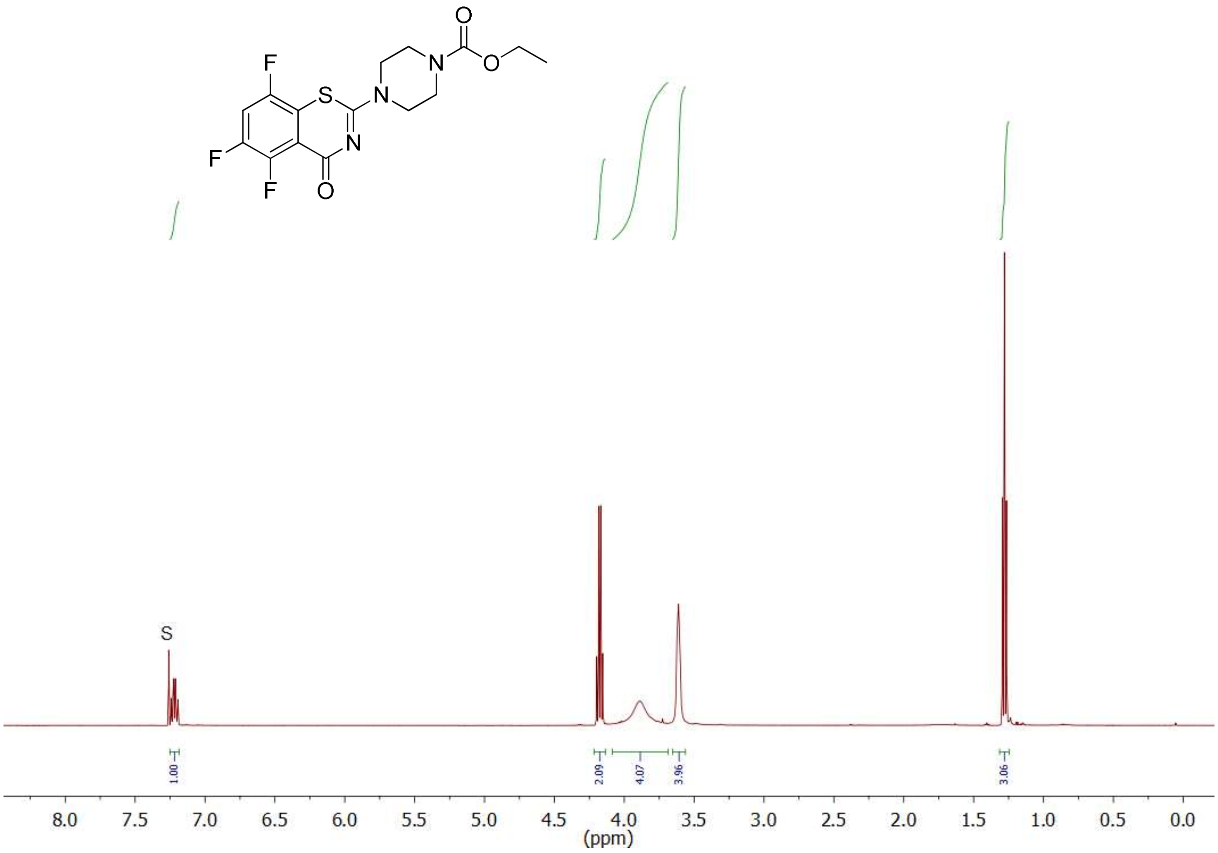

Figure S7 ^{1}H NMR spectrum of **2c** in chloroform-*d*. S denotes the solvent peak.

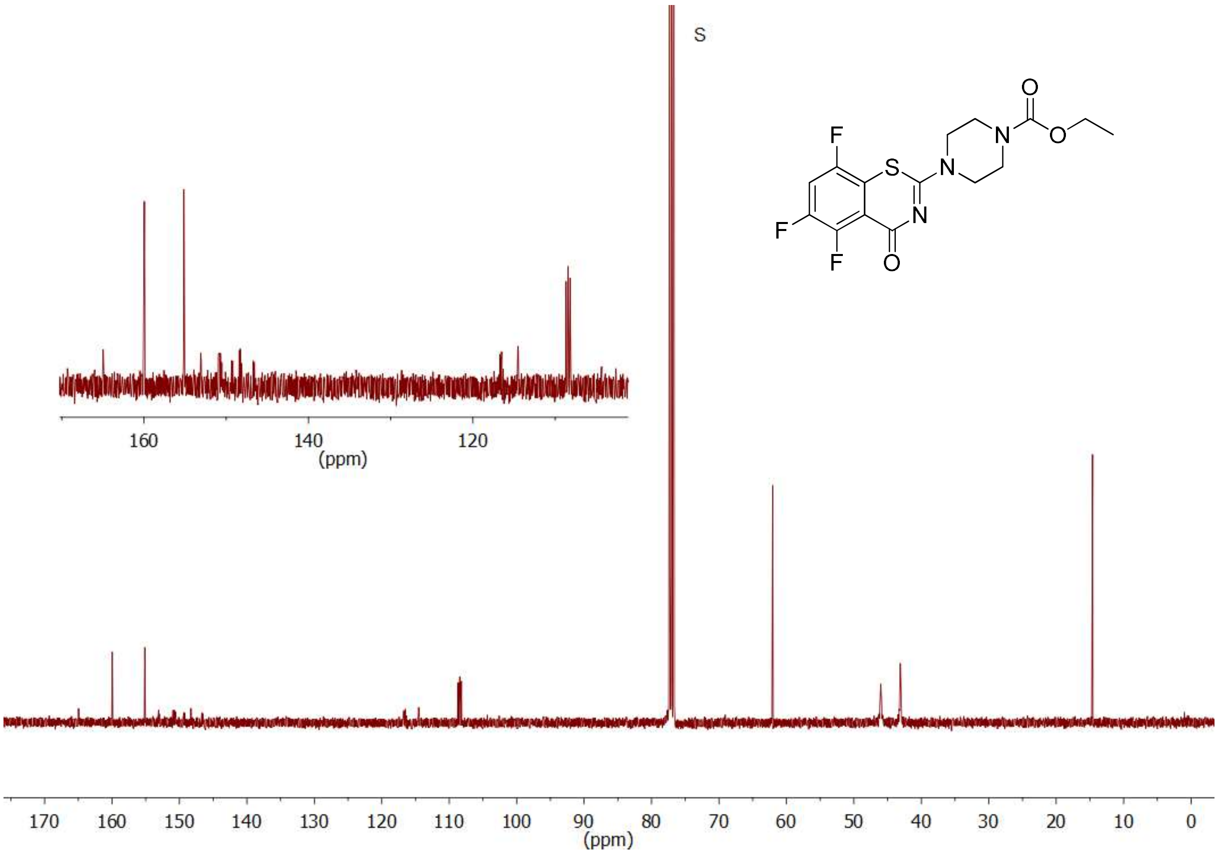

Figure S8 ^{13}C{^{1}H} NMR spectrum of **2c** in chloroform-*d*. S denotes the solvent peak.

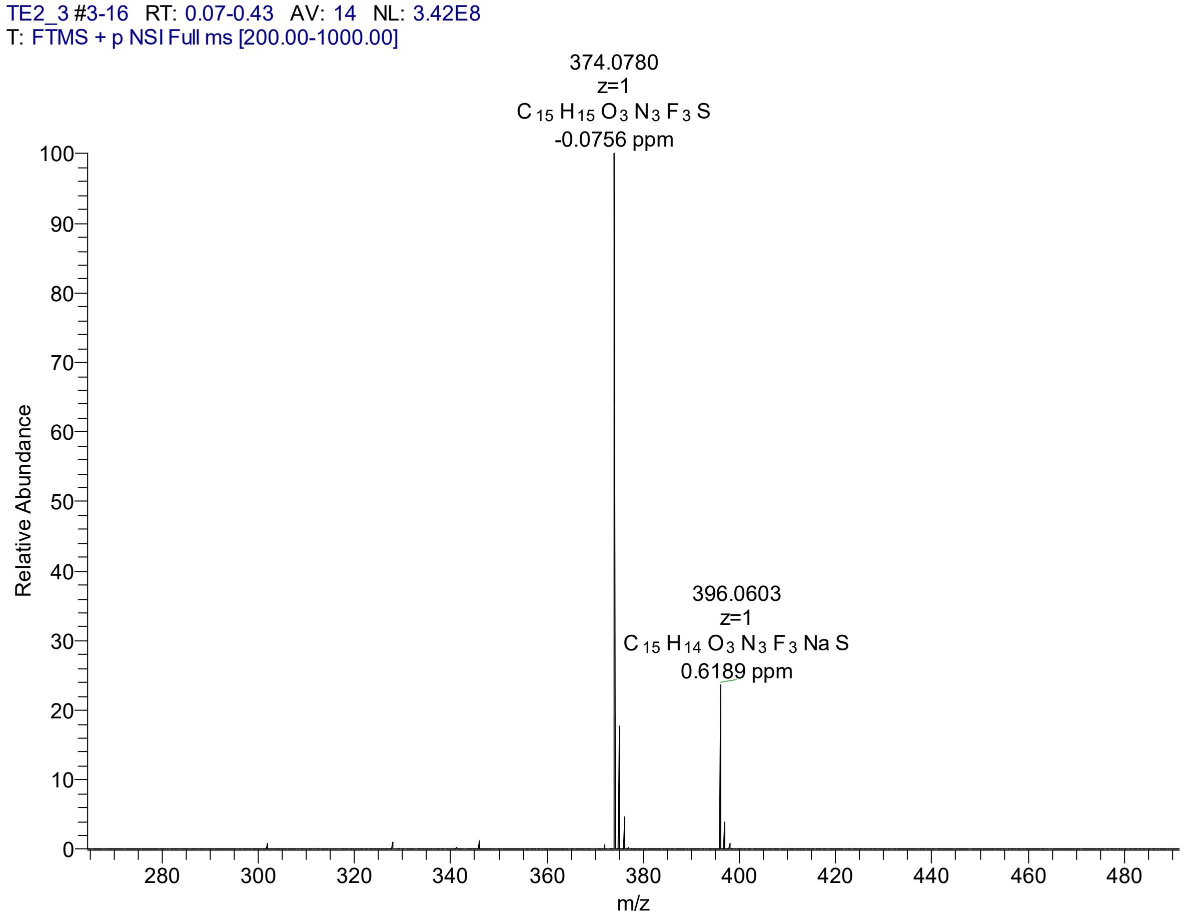

Figure S9 HRMS(ESI$^+$) spectrum of **2c** in methanol.

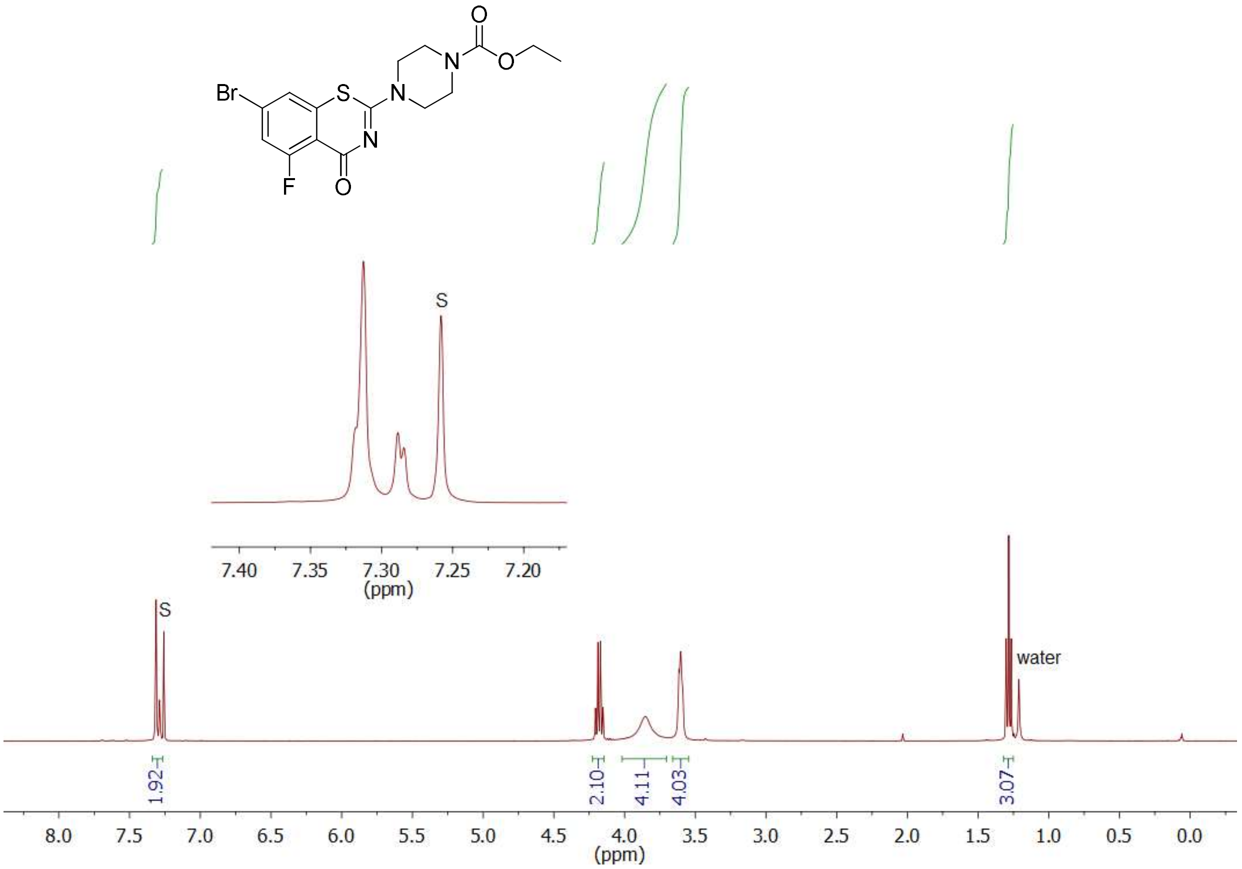

Figure S10 ^{1}H NMR spectrum of **2d** in chloroform-*d*. S denotes the solvent peak.

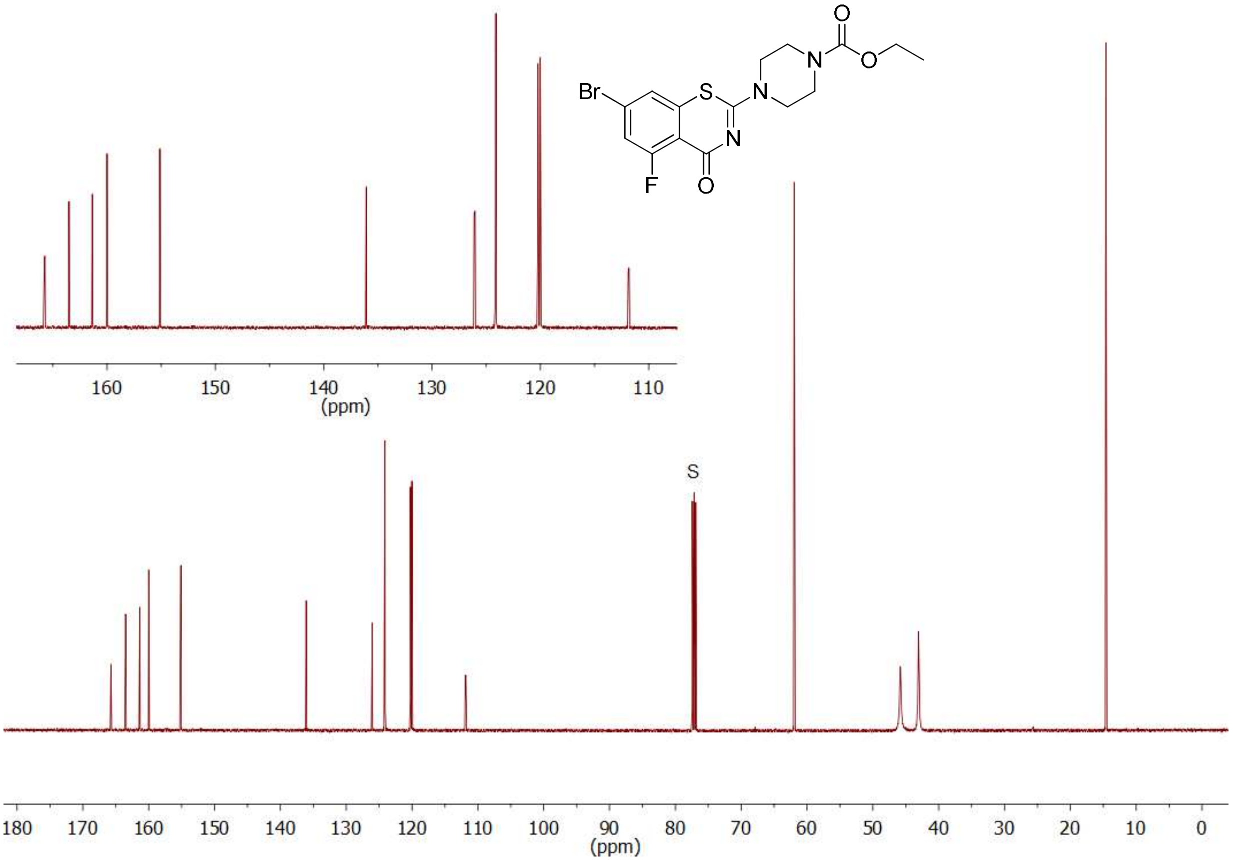

Figure S11 ^{13}C{^{1}H} NMR spectrum of **2d** in chloroform-*d*. S denotes the solvent peak.

```
electrospray-ionization (Sol.: CH3OH )  pos. ions
molecular weight 376, 415 et. al. possible
characteristical ions
377 = [376 + H]+
399 = [376 + Na]+
416 = [415 + H]+
438 = [415 + Na]+
853 = [2*415 + Na]+
```

```
10.02.2021
File: 151613a-00.RAW
Analyse: GOD-GB-078-01
LMN: Goddard,Richard
Ionisierung:  ESIpos
Lösungsmittel:CH3OH
Spektrometer: Exactive
Auswerter:    Kampen (2242)
```

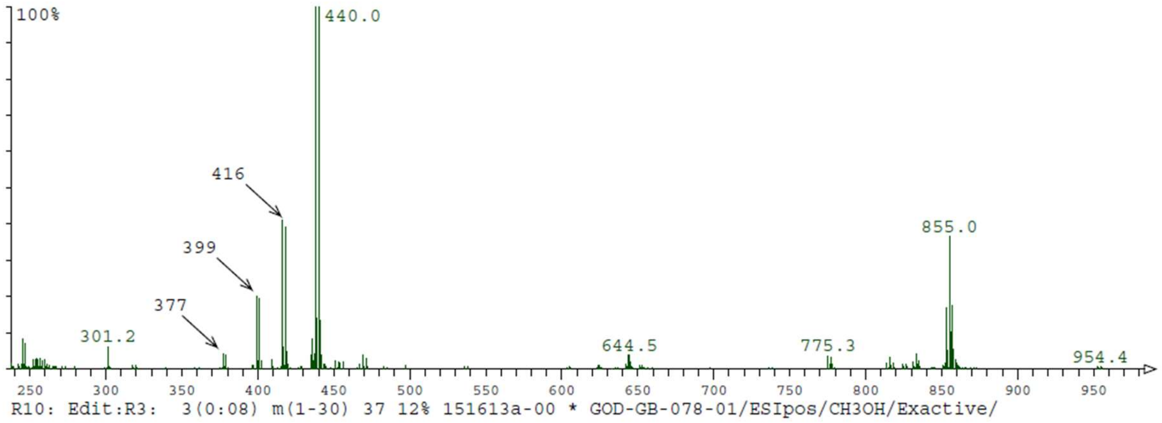

R10: Edit:R3: 3(0:08) m(1-30) 37 12% 151613a-00 * GOD-GB-078-01/ESIpos/CH3OH/Exactive/

Mass to be matched (m/z): 437.989650 Charge: 1

Mass Tolerance: ±0.003000

Restriction of atom numbers:

C	H	Br	F	N	O	S	Na
1-100	1-100	1-1	max 1	1-3	max 10	max 1	1-1

Number of calculated Formulas: 9

Formula	Diff.(ppm)	theor. m/z
C15 H15 Br1 F1 N3 O3 S1 Na1	-0.60	437.989386
C23 H11 Br1 F1 N1 O1 Na1	0.88	437.990036
C26 H10 Br1 N1 Na1	-1.73	437.988893
C14 H14 Br1 N3 O7 Na1	2.50	437.990745
C18 H14 Br1 N3 O2 S1 Na1	-3.21	437.988243
C11 H16 Br1 N2 O10 Na1	-3.62	437.988066
C11 H15 Br1 F1 N3 O8 Na1	5.11	437.991889
C23 H14 Br1 N1 S1 Na1	5.97	437.992265
C12 H17 Br1 F1 N2 O6 S1 Na1	-6.72	437.986708

```
Datum            10.02.2021
Analyse:         151613b-00
Sigel:           GOD-GB-078-01
LMN: Goddard,Richard
Messung:         HRMS
Methode:         ESIpos
Lösungsmittel:   CH3OH
Spektrometer:    Exactive
Auswerter:       Kampen (2242)
```

```
Suggestion:
C15H15Br1F1N3O3S1  MW 415

characteristical ion
438 = [415 + Na]+
```

Figure S12 HRMS(ESI$^+$) analysis of **2d** in methanol.

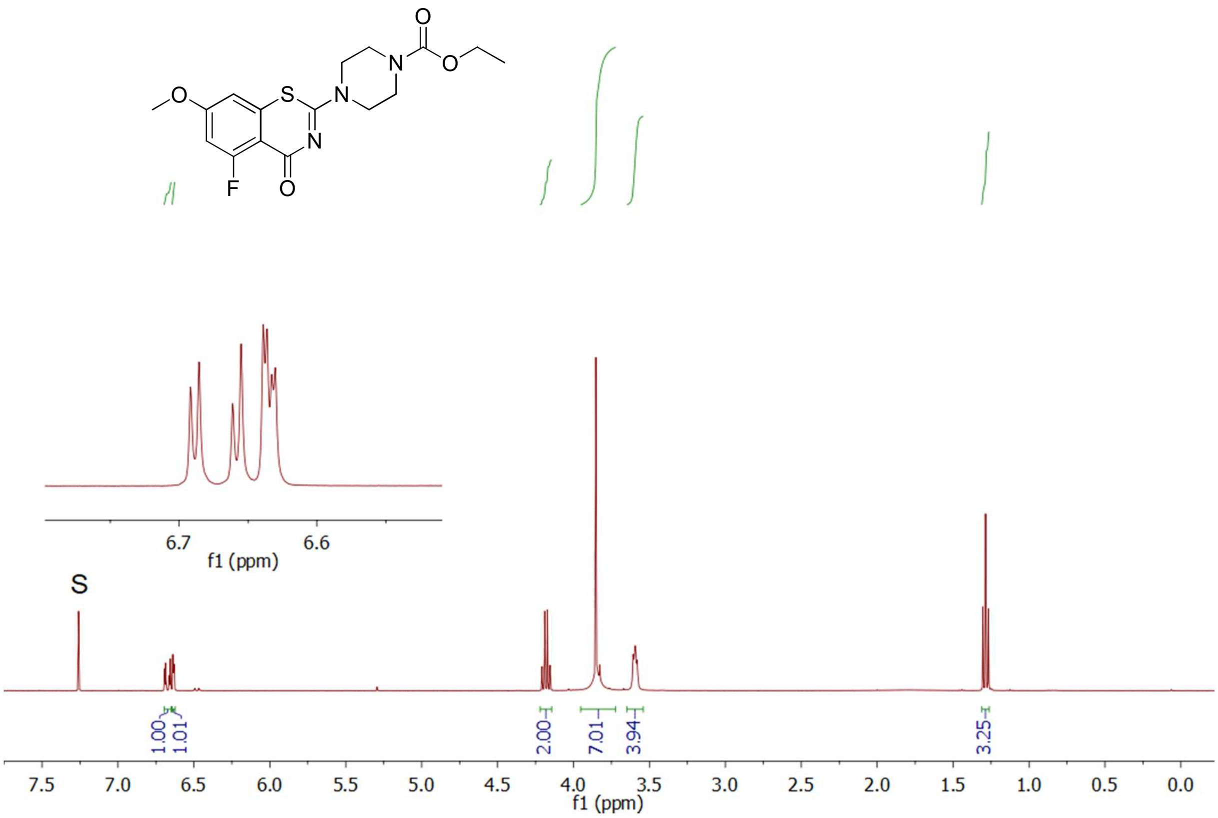

Figure S13 ^{1}H NMR spectrum of **2e** in chloroform-*d*. S denotes the solvent peak.

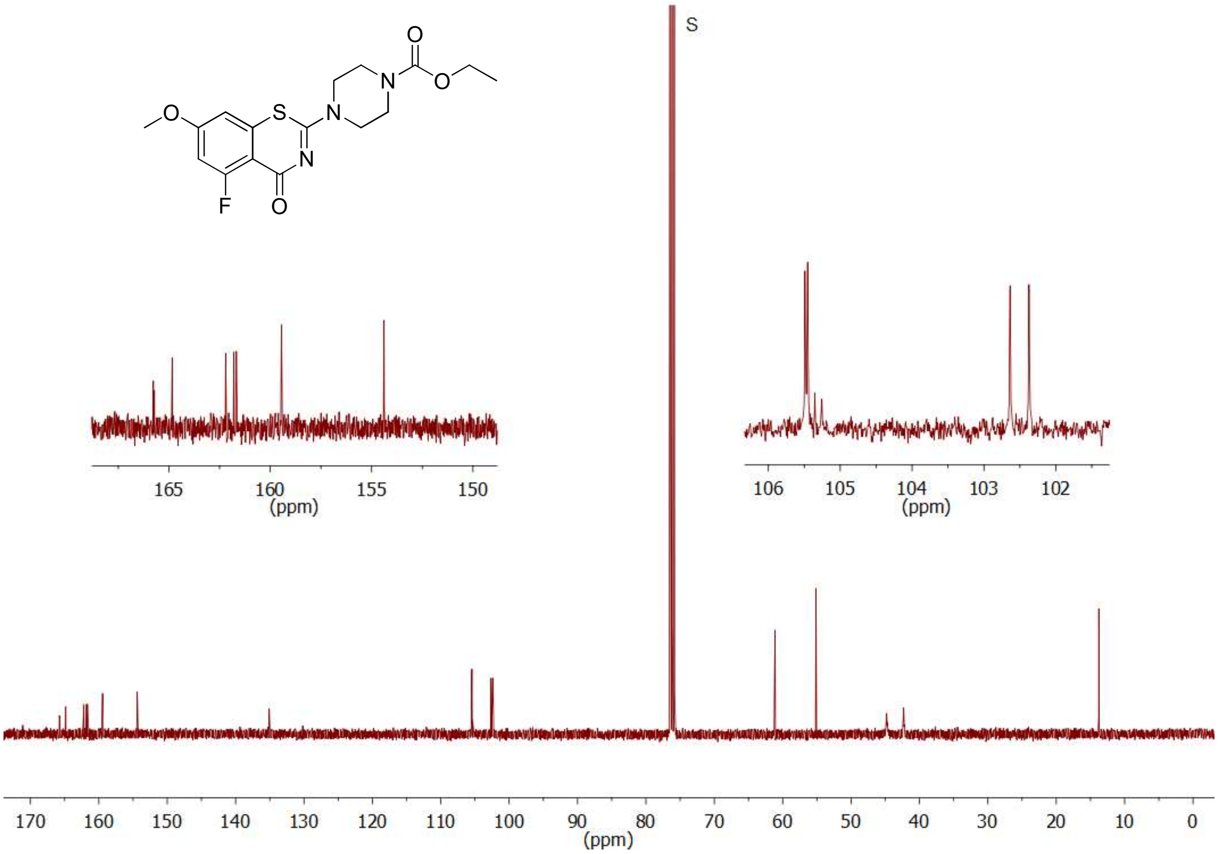

Figure S14 ^{13}C{^{1}H} NMR spectrum of **2e** in chloroform-*d*. S denotes the solvent peak.

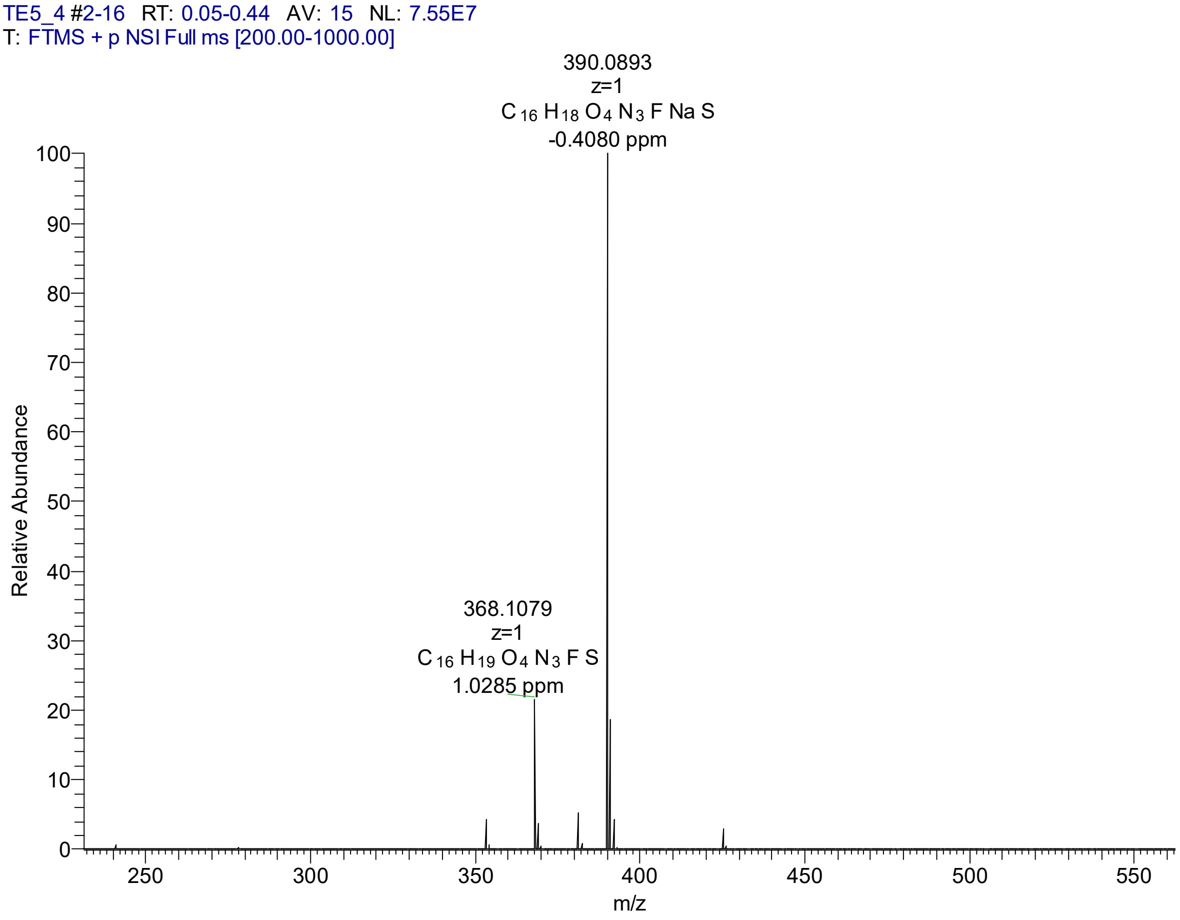

Figure S15 HRMS(ESI$^+$) spectrum of **2e** in methanol.
